# A rare case of congenital cystic adenomatoid malformation: Mimics pneumonia manifestations

**DOI:** 10.1016/j.amsu.2021.102433

**Published:** 2021-05-27

**Authors:** Maysaa Badour, Bara'a Hussain, Ali Hammed, Sawssan ali, Saeed falyon

**Affiliations:** a Pediatric University Hospital, Damascus, Syria; b Tishreen University Hospital, Department of Neurosurgery. Lattakia, Syria

**Keywords:** Congenital cystic adenomatoid malformation, Pneumonia, Lobectomy

## Abstract

**Introduction and importance:**

Congenital cystic adenomatoid malformation (CCAM) is a rare malformation, with unknown causes, that affects distal bronchi. It accounts for 25% of congenital pulmonary malformation s and most cases are found in neonates and babies.

The mortality rate of prenatally diagnosed cases ranges from 9 to 49%. The risk factors for poor outcome include hydropsfoetalis, microcystic CCAM and the overall size of the lesions.

The mainstay of CCAM treatment is surgical excision that prevents complications such as recurrent infections, pneumothorax and malignancy.

**Case presentation:**

Our case is a 4-month-old boy born presented with shortness of breath and poor suckling. He was admitted to intensive care for respiratory distress.

**Clinical discussion:**

A working diagnosis of pneumonia was entertained and the patient given humidified oxygen through nasal prongs, intravenous fluids and antibiotics.

Thoracic CT showed a cystic malformation in the left lower lung lobe.

Due to continuing recurrent infections and the risk of rupturing of the cyst with subsequent pneumothorax, it was decided to proceed with a left lower lobectomy.

The post-operative course was uneventful and the patient was discharged home on the fifth post-operative day.

**Conclusion:**

Congenital cystic adenomatoid malformation should be a differential diagnosis of pneumonia.

A real awareness of this rare entity among pediatricians and radiologists should allow early diagnosis and proper treatment.

## Highlights

• Congenital cystic adenomatoid malformation (CCAM) is a rare malformation, with unknown causes, that affects distal bronchi.
• Congenital cystic adenomatoid malformation should be a differential diagnosis of pneumonia.
• A real awareness of this rare entity should allow early diagnosis and proper treatment.

## 1 Introduction

Congenital Cystic Adenomatoid Malformation CCAM is a rare abnormality of lung development [1].

Congenital cystic adenomatoid malformation (CCAM) is a rare malformation, with unknown causes, that affects distal bronchi [1]. It accounts for 25% of congenital pulmonary malformation s and most cases are found in neonates and babies. It yields single or multiple cyst s in different locations, with ipsilateral or even mediastinal pulmonary compression [2].

The incidence of prenatally diagnosed CCAM is 1: 25,000–35,000. CCAM may present in old children and adults as incidental finding secondary to repeated infection [3].

The mortality rate of prenatally diagnosed cases ranges from 9 to 49%. The risk factors for poor outcome include hydropsfoetalis [4,5], microcystic CCAM [4,6] and the overall size of the lesions [4,7,8].

Currently, thanks to advances in prenatal imaging, pulmonary tract defects can be detected during pregnancy or at birth [9].

The imagery is based primarily on CT scan, but the diagnosis remains difficult because of its scarcity [10].

The mainstay of CCAM treatment is surgical excision that prevents complications such as recurrent infections, pneumothorax and malignancy [3,11].

Our work is a single case report and has been reported in line with the SCARE criteria [12].

## 2 Case report

A 4-month-old boy born via a normal pregnancy without complications.The mother was on regular antenatal care but she did not have antenatal ultrasound examination. The baby had an Apgar score of 9 and 10 at one and 5 min. There weren't any congenital abnormalities. He was discharged home after 2 h of delivery.

He had had several episodes of respiratory distress and pulmonary infections, which were successfully treated with antibiotics.

He presented with shortness of breath and poor suckling. He was admitted to intensive care for respiratory distress. Physical examination showed an ill looking baby with central cyanosis, tachypnea (respiratory rate of 55),.intercostal and subcostal recessions with course right basal crackles. Laboratory tests were unremarkable.

The patient's review of systems and additional medical history surgical, family, psychosocial and pharmacologic were unremarkable.

A working diagnosis of pneumonia was entertained and the patient was admitted to intensive care unit, given humidified oxygen through nasal prongs, intravenous fluids and antibiotics.

**Fig. 1:**
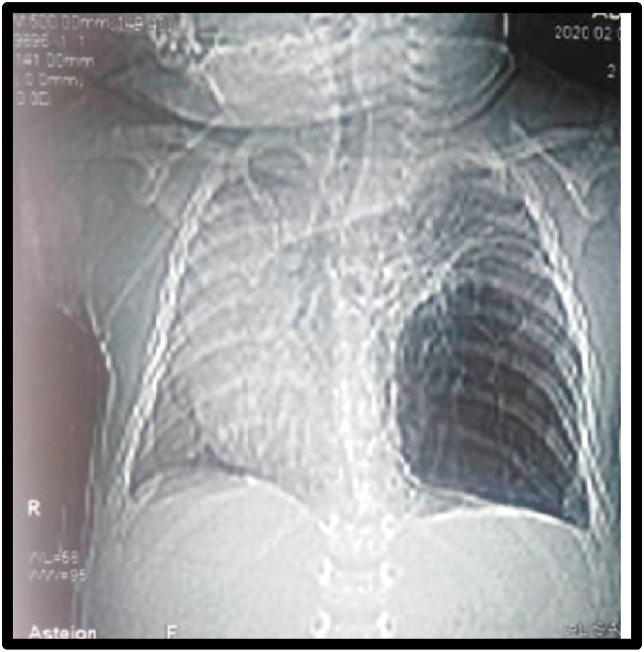
Initial chest x ray shows hyper-lucency of the affected lower left lobe with midline and heart shift to the opposite side and compressive atelectasis of adjacent ipislateral lung lobes.

Initial chest x ray showed hyper-lucency of the affected lower left lobe with midline and heart shift to the opposite side and compressive atelectasis of adjacent ipislateral lung lobes (Fig. 1).

**Fig. 2:**
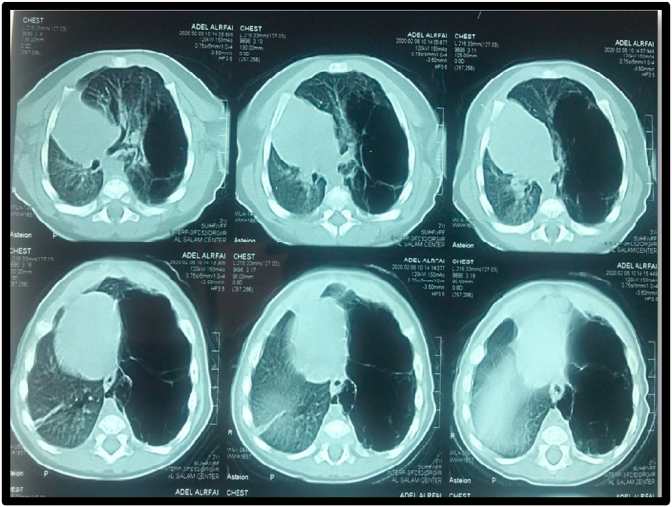
Thoracic CT showed a cystic malformation in the left lower lung lobe. **It reveals a large cystic lesion with well defined wall in left** lower lung lobe **consist of multiple cysts of varying size (3**–**6 cm).**

Thoracic CT showed a cystic malformation in the left lower lung lobe. **It revealed a large cystic lesion with well defined wall in left** lower lung lobe **consist of multiple cysts of varying size (3**–**6 cm).** Fig. 2.

Due to continuing recurrent infections and the risk of rupturing of the cyst with subsequent pneumothorax, it was decided to proceed with a left lower lobectomy.

After obtaining the parents' informed consent, surgery was planned.

The procedure was done by a consultant surgeon.

**Fig. 3:**
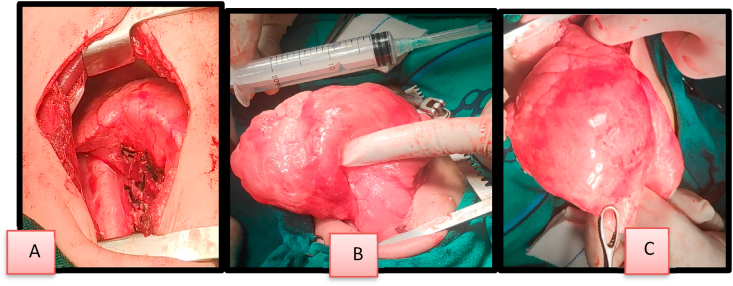
(A-B-C) Intraoperation shows resection of left lower lobe.

The patient underwent an uneventful left lower lobectomy via a posterolateral thoracotomy. The gross specimen was 7*4*5 cm (Fig. 3).

Histopathology showed that the lung parenchyma exhibited variable-sized interconnecting cystic spaces resembling bronchioles and lined by bronchial-type epithelium. Smooth muscle bundles, a few areas with hemorrhage, and macrophages were also seen, and the intervening lung parenchyma showed alveolar ducts and underdeveloped alveoli.

These features were consistent with a CCAM - Type 1.

The post-operative course was uneventful and the patient was discharged home on the fifth post-operative day.

**Fig. 4:**
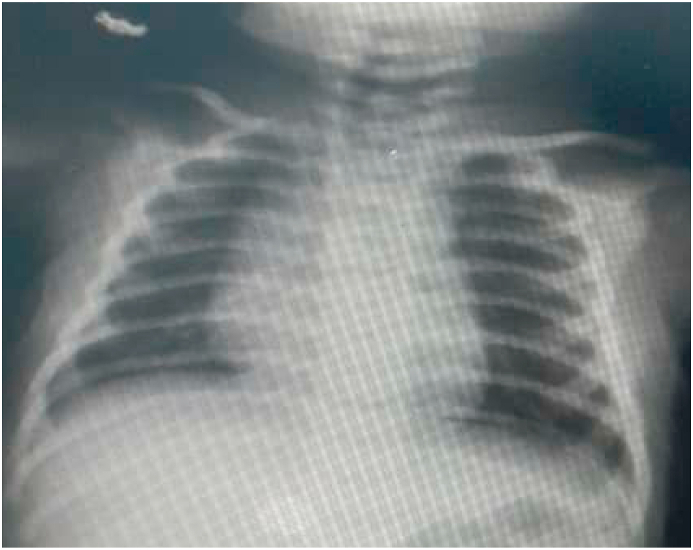
Follow-up X-ray after 20 days of operation shows normal findings.

Follow-up chest Xray taken on postoperative day 20 and showed normal findings (Fig. 4).

After one year of operation, He has remained asymptomatic on follow-up clinical examinations and chest X-rays at our clinic every 3 months.

## 3 Discussion

CCAM is a rare developmental, non-hereditary, hamartomatous abnormality of the lung of unknown cause. First described in 1949 by Ch'in and Tang [13].

The pathogenesis of CCAM is uncertain. The primary defect during development that leads to CCAM has been described as either bronchial atresia or maturation arrest in bronchopulmonary segments before the 17th week of gestation. The morphology of the lesion is then determined by the dysplastic lung growth beyond the atreticsegment [14,15].

The prenatal diagnosis can usually be established by ultrasonography. Up to 71% of such cases are asymptomatic at birth, while spontaneous regression has been reported in up to 76% of cases without any prenatal intervention [16,17].

CCAM is often misdiagnosed by radiology a s a pulmonary cyst, bubbles of emphysema, or pneumothorax [18].

Diagnosis of CCAM is accomplished by imaging studies but only histopathology is confirmatory. Chest x-ray may show a mass containing air filed cysts [19].

CT scan provides a more detailed anatomy. The typical appearance is of multiocular cystic lesions with thin walls surrounded by normal lung parenchyma. The presence of superimposed infecting with the lesion may complicate the appearance [20].

Chest radiographs can suggest a localized patchy density, namely a cysti mass; butMDCT best demonstrates the cy stic and solid components while ruling out bronchiectasis or a major bronchial obstruction.

Stocker JT et al. [21] published a classification of CCAM which later revised by Stocker in Ref. [22] 2002 and this is currently the most acceptable classification used for diagnosis and treatment planning.

Up to 26% of cases can be associated with other congenital anomalies, including extralobular sequestration, diaphragmatic hernia, pulmonary hypoplasia and cardiovascular malformations. In general, lungs with CCAM have a normal arterial supply and venous drainage, although anomalous vascular communications have been reported [23].

Apart from the mentioned complications, the big risk of CC A M is to develop a bronchioloalveolar carcinoma or other type of malignant transformation for example, sa rcoma, or blastoma [24].

The evolution of CCAM surgery is usually favorable. It leads to a low rate of postoperative morbidity and mortality, shorter hospital stay, and mainly lowers the risk of recurrence [1,7,10,25]. It can lead to more or less severe respiratory failure [25,26].

## 4 Conclusion

Congenital cystic adenomatoid malformation should be a differential diagnosis of pneumonia.

A real awareness of this rare entity among pediatricians and radiologists should allow early diagnosis and proper treatment, avoiding the use of antibiotics, antituberculosis drugs, and chest drainage, which can be dangerous.

## Sources of funding

This research did not receive any specific grant from funding agencies in the public, commercial, or not-for-profit sectors.

## Ethical approval

This study was not applicable for ethical approval.

## Consent

A written informed consent was obtained from the patient's parents for publication of this Case report and accompanying images.

## Registration of research studies

The case report at hand is not a first-in-man case report of a novel technology or surgical technique, therefore a registration of these case reports according to Declaration of Helsinki 2013 is not required.

### Provenance and peer review

Not commissioned, externally peer-reviewed.

## Declaration of competing interest

All authors declared no conflict of interest.
